# PSA im Extrembereich – Ausdruck einer infausten Prognose? Eine Übersicht über Verlauf und Therapie des Prostatakarzinoms mit PSA-Werten (prostataspezifisches Antigen) im drei- bis vierstelligen Bereich

**DOI:** 10.1007/s00120-025-02583-9

**Published:** 2025-05-19

**Authors:** Christian Dirscherl, Thomas Ebert, Bernd J. Schmitz-Dräger, Peter J. Goebell

**Affiliations:** Uniklinikum Erlangen und Friedrich‐Alexander‐Universität Erlangen‐Nürnberg (FAU), Krankenhausstraße 12, 91054 Erlangen, Deutschland

**Keywords:** Prostatakarzinom, Prostataspezifisches Antigen, Prognose, Real world, Database, Prostate neoplasms, Prostate-specific antigen, Prognosis, Real world, Database

## Abstract

**Hintergrund:**

Extrem hohe Baseline-PSA-Werte (bPSA) im Bereich von 100 bis ≥ 1000 ng/ml vor Beginn der systemischen Therapie stellen eine Herausforderung dar, da der Eindruck einer sehr ungünstigen Prognose entstehen kann.

**Ziel der Arbeit:**

Wir untersuchten Einflussfaktoren auf das 5‑Jahres-Gesamtüberleben (5-JGÜ) und die Therapiemodalitäten von Betroffenen mit bPSA-Werten von ≥ 100 ng/ml anhand retrospektiver Daten.

**Material und Methoden:**

Aus einem kleineren initialen Kollektiv legten wir Items fest, die wir für eine Abfrage aus der Datenbank UroCloud nutzten. Dabei wurden insgesamt 695 Betroffene eingeschlossen.

**Ergebnisse und Schlussfolgerung:**

Für das gesamte Kollektiv ergab sich ein 5‑JGÜ von 68,5 % ± 2,7 %. Der bPSA-Wert hatte in folgender Gruppierung signifikanten (*p* < 0,001) Einfluss auf das 5‑JGÜ: 100–149 ng/ml, 150–249 ng/ml, 250–649 ng/ml, ≥ 650 ng/ml (5-JGÜ: 77,0 % ± 4,9 % vs. 76,9 % ± 4,5 % vs. 61,4 % ± 6,0 vs. 57,4 %± 6,1 %). Ein Alter von ≤ 70 Jahren gegenüber > 70 Jahren bei Erstdiagnose ergab einen signifikanten Unterschied bezüglich des 5‑JGÜ (5-JGÜ: 74,8 % ± 3,5 % vs. 60,1 % ± 4,4). Eine PSA-Response von > 90 %, die in 79,0 % der Fälle erreicht wurde, hatte signifikanten Einfluss auf das 5‑JGÜ gegenüber einer Response von ≤ 90 % (5-JGÜ: 73,5 % ± 2,9 % vs. 48,6 % ± 6,7 %). Ebenso zeigte sich ein signifikanter Vorteil im 5‑JGÜ, wenn ein erster PSA-Nadir von < 0,20 ng/ml erreicht wurde (in 22,2 % der Fälle), gegenüber einem ersten PSA-Nadir von ≥ 0,20 ng/ml (5-JGÜ: 89,8 % ± 3,3 % vs. 60,4 % ± 3,5 %). In 49,4 % (*n* = 343) der Fälle wurde als systemische Erstlinientherapie eine Androgendeprivationstherapie (ADT) als Monotherapie begonnen, über den Zeitraum 1999–2023 sahen wir eine Zunahme an gestaffelten und initialen Kombinationstherapien. Die Untersuchung zeigt, dass ein extremer bPSA-Wert für sich kein Ausdruck einer ungünstigen Prognose ist.

**Graphic abstract:**

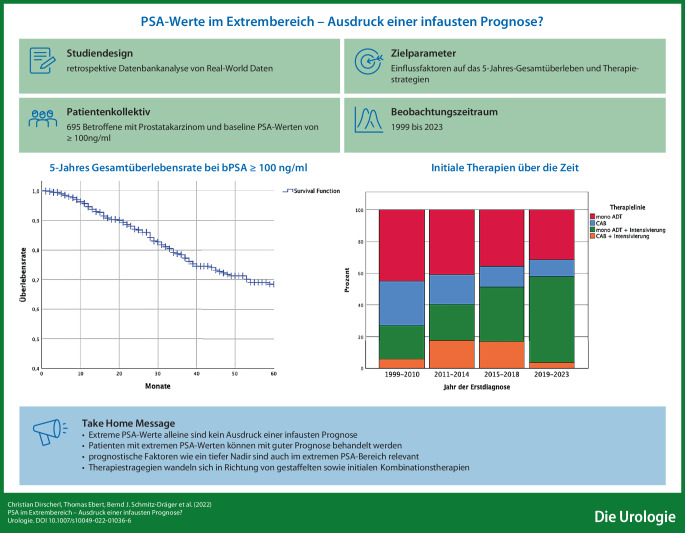

## Abstract (en)

**Background:**

Extremely high baseline prostate-specific antigen (bPSA) values in the range of 100 to ≥ 1000 ng/ml prior to the start of systemic therapy pose a challenge, as they can lead to the impression of a very unfavorable prognosis.

**Objective:**

We investigated factors influencing 5‑year overall survival (5-yOS) and the treatment modalities of patients with bPSA levels ≥ 100 ng/ml using retrospective data.

**Materials and methods:**

We defined items from a limited initial collective, which we then used for a query in the UroCloud database. A total of 695 patients were included.

**Results and conclusion:**

For the entire collective, the 5‑yOS was 68.5% ± 2.7%. The bPSA value had a significant (*p* < 0.001) influence on 5‑yOS in the following groups: 100–149 ng/ml, 150–249 ng/ml, 250–649 ng/ml, and ≥ 650 ng/ml (5-yOS: 77.0% ± 4.9% vs. 76.9% ± 4.5% vs. 61.4% ± 6.0 vs. 57.4% ± 6.1%, respectively). Age ≤ 70 years compared to > 70 years at first diagnosis resulted in a significant difference regarding 5‑yOS (74.8% ± 3.5% vs. 60.1% ± 4.4, respectively). A PSA response of > 90%, which was achieved in 79.0% of cases, had a significant influence on 5‑yOS compared to a response of ≤ 90% (73.5% ± 2.9% vs. 48.6% ± 6.7%, respectively). There was also a significant 5‑yOS advantage if a first PSA nadir of ≤ 0.20 ng/ml was achieved (in 22.2%) compared to a first PSA nadir of > 0.20 ng/ml (89.8% ± 3.3% vs. 60.4% ± 3.5%, respectively). In 49.4% (*n* = 343) of cases, androgen deprivation therapy (ADT) monotherapy was started as first-line systemic therapy. Between 1999 and 2023, we saw an increase in escalated and initial combination therapies. The analysis shows that an extreme bPSA value is not in itself an expression of an unfavorable prognosis.

## Hinführung zum Thema

Der PSA-Wert ist untrennbar mit der Diagnostik und Behandlung des Prostatakarzinoms (PCa) verbunden: als Wegweiser in der Früherkennung, zur Kontrolle des Therapieansprechens oder auch bei der Detektion eines Rezidivs nach definiter Therapie. Extreme PSA-Werte von 100 bis ≥ 1000 ng/ml zu Beginn einer systemischen Therapie stellen eine diagnostische und therapeutische Herausforderung dar, da wenig über ihre prognostische Relevanz bekannt ist und der Eindruck einer ungünstigen Prognose mit kurzem Überleben entsteht.

Wir führten eine retrospektive Untersuchung an 695 Betroffenen durch, die einen PSA-Wert von ≥ 100 ng/ml vor Beginn der systemischen oder in seltenen Fällen lokalen Therapie aufwiesen.

## Hintergrund und Fragestellung

Das PCa stellt die Krebserkrankung mit der höchsten Neuerkrankungsrate und die zweithäufigste Ursache für krebsbedingte Todesfälle bei Männern in Deutschland dar [26]. Somit betrifft das PCa eine Vielzahl an Individuen, die ebenso viele individuelle Krankheitsverläufe erfahren. Um den Betroffenen eine auf den Einzelfall bestmöglich abgestimmte Therapie anzubieten, sind diagnostische Parameter nötig, die es ermöglichen, den Erkrankungsverlauf darzustellen und damit die Therapiestrategie und Therapieziele gemeinsam mit dem Patienten festzulegen. Einer dieser Parameter ist der PSA-Wert im Serum. Neben seiner Verwendung als Parameter in der Früherkennung wird er auch zur Kontrolle des Therapieansprechens oder bei der Detektion eines Rezidivs nach definiter Therapie eingesetzt. Meist sind die Werte, anhand derer Entscheidungen getroffen werden, Grenzwerte im niedrigen einstelligen Bereich.

Neben der reinen Höhe des PSA-Wertes spielt sowohl die Kinetik, wie z. B. die PSA-Verdoppelungszeit, als auch der reine Betrag der Veränderung in der Diagnostik eine Rolle. Es wurden verschiedene Konzepte erarbeitet, um aus diesen Veränderungen eine Aussage über den Status der Erkrankung zu ermöglichen. Der Betrag des PSA im Serum wird zum Zeitpunkt der Erstdiagnose in Form von Grenzwerten eingeordnet. Hier ist v. a. die Erhöhung auf ≥ 4 ng/ml zu erwähnen, die, in der Zusammenschau mit anderen Befunden, den Weg zu weiterer Diagnostik weist [19]. Auch neuere Untersuchungen zur Früherkennung nehmen den PSA-Wert als Entscheidungshilfe heran, wenn es um die weitere Planung nachfolgender Untersuchungen und deren zeitliche Einordnung geht [2].

Bereits die Mitteilung eines Messwertes von ≥ 4 ng/ml im einstelligen Bereich kann auf Betroffene wie die definitive Diagnose einer schwerwiegenden Krebserkrankung wirken. In einer Befragung durch Biddle et al. wird deutlich, dass Betroffene nach der Mitteilung einer solchen Erhöhung einen linearen Zusammenhang zwischen Erhöhung und Erkrankungsrisiko annehmen [5]. Bei Konfrontation mit einer Erhöhung des PSA-Wertes um das mehr als 100Fache der Grenzwerte, die in den Leitlinien definiert sind, kann so der Eindruck einer sehr negativen Prognose entstehen. In anderen Untersuchungen konnte gezeigt werden, dass ein PSA-Wert von > 100 ng/ml mit einer schlechteren Prognose bezüglich des Überlebens einhergeht [1, 16]. Es zeigt sich, dass es eine starke Korrelation zwischen der Höhe des PSA-Wertes und dem Ausmaß der Angst und des Distress gibt, die Patienten im extremen PSA-Bereich erleben [27]. Hier ist die Aufgabe der Behandelnden, gemeinsam mit den Betroffenen ein individuelles Therapiekonzept zu entwickeln und eben auch deren Sorgen und Ängsten zu begegnen. Um hier eine reelle Einschätzung der Erkrankungssituation und -perspektive vorzunehmen, sind klinische Daten bezüglich des zu erwartenden Verlaufes und dem möglichen Erfolg der Therapie bei extremen PSA-Werten notwendig.

Bei der Suche nach Handlungsempfehlungen zu deutlich über ≥ 100 ng/ml erhöhten Werten findet sich in der S3-Leitlinie Prostatakarzinom lediglich die Erhöhung > 50 ng/ml als Anhaltspunkt für das sofortige Einleiten einer hormonablativen Therapie, um der ab diesem Wert nachgewiesenen deutlich erhöhten Mortalität zu begegnen [19]. Auch in den Zulassungsstudien wie der LATTITUDE, CHAARTED oder STAMPEDE finden sich im Patientenkollektiv mediane PSA-Werte von 23,8 ng/ml, 50,9 ng/ml bis hin zu 65 ng/ml von Patienten mit bestehenden Metastasen und hoher Krankheitslast [15, 22, 31]. Dadurch liegen zwar Informationen zum Bereich von PSA-Werten bis 100 ng/ml vor, doch sobald dieser Bereich aber überschritten wird, können keine differenzierten Einschätzungen bezüglich der Therapie und Prognose aus diesen großen Studien abgeleitet werden.

In dieser Arbeit untersuchten wir retrospektiv Patientendaten aus der Datenbank des Uniklinikums Erlangen, ambulant tätiger Urologen sowie aus der Datenbank UroCloud mit Baseline-PSA-Werten (bPSA) ≥ 100 ng/ml. Dabei liegt unser Augenmerk primär auf dem 5‑Jahres-Gesamtüberleben (5-JGÜ) nach Erstdiagnose sowie der angewandten Therapie und deren mögliche Intensivierung, wobei wir einen Überblick über die in unserem Kollektiv verbreiteten Therapieansätze geben.

## Studiendesign und Untersuchungsmethoden

### Kollektiv

**Abb. 1:**
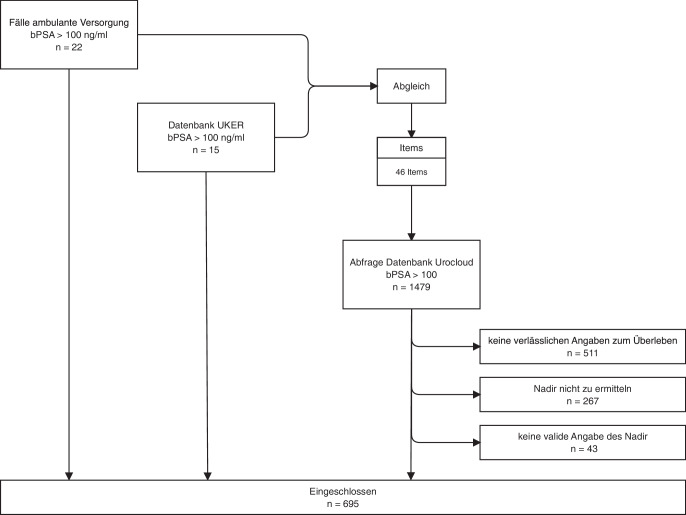
CONSORT

In unserer retrospektiven Analyse nutzen wir einen bPSA-Wert von ≥ 100 ng/ml als primäres Einschlusskriterium. Zunächst untersuchten wir 22 Fälle als Trainingsset, die uns unsere ambulanten Partner anonymisiert zur Verfügung stellten, um hieraus die zu untersuchenden vorläufige Analyseitems zu generieren, die wir mit den Daten von 15 Fällen aus der klinischen Datenbank des Universitätsklinikums Erlangen abglichen. Aus diesem Abgleich gingen schlussendlich 46 Analyseitems hervor, die dann in die Datenbankabfrage aus der UroCloud mündeten. Die UroCloud ist eine prospektive Datensammlung, die seit 2005 besteht und an der sich mehr als 400 teilnehmende Behandelnde aus 40 teilnehmenden Kliniken beteiligen. Sie enthält insgesamt Daten zu 32.700 an einem PCa erkrankten Patienten, woraus sich eine Betrachtung von 95.000 Patientenjahren ergibt, bei mindestens einem Kontakt pro Jahr. Seit September 2023 erfolgt die Bereitstellung und Weiterentwicklung der UroCloud (jetzt als UroCloud^plus^) durch die VgURO (Versorgungsgesellschaft Urologie mbH) ein Tochterunternehmen des BvDU (Berufsverband der Deutschen Urologie e. V.; [4]). Der Kreis der teilnehmenden Kliniken wird somit auch erweitert werden können. Aus der UroCloud-Abfrage resultierten 1479 Fälle, die zusammen mit dem Trainingskollektiv die Grundgesamtheit des Untersuchungskollektivs bildeten, wie im CONSORT-Diagramm hinterlegt. In 511 Fällen konnte keine verlässliche Angabe zum Überleben ermittelt werden, in 267 Fällen lagen keine validen Informationen zum PSA-Nadir vor und in 43 Fällen war der angegeben Wert des Nadirs nicht plausibel. Hieraus ergeben sich 695 eingeschlossene Fälle. Hiervon wurden 64,0 % (*n* = 445) der Fälle an zu diesem Zeitpunkt durch den DVPZ (Dachverband der Prostatazentren Deutschlands e. V. – seit Oktober 2022 URO-Cert-Verband urologischer Kompetenzzentren e. V.) zertifizierte Zentren erhoben. Der Baseline-PSA-Wert (bPSA) entspricht in der Regel dem PSA-Wert vor Beginn der systemischen Therapie. In seltenen Fällen (*n* = 39) auch dem PSA-Wert vor einer definiten lokalen Therapie, die trotz der extremen PSA-Werte eingeleitet wurde (Abb. 1).

### Erhobene Parameter

Die aus den eingeschlossenen Patientenakten erhobenen Analyseitems umfassen u. a. das Alter bei Erstdiagnose, den bPSA-Wert, den zeitlichen Verlauf des PSA-Wertes einschließlich des ersten PSA-Nadirs und des jemals erreichten PSA-Nadirs, den Gleason Score, das UICC-Stadium (Union Internationale Contre le Cancer), die karzinomgerichtete Therapie im zeitlichen Verlauf sowie den Grad der Metastasierung. Das Überleben der Patienten wurde mittels Kaplan-Meier-Analyse als 5‑JGÜ ermittelt.

### Statistische Analyse

Für die statistische Analyse wurde die Überlebensanalyse nach Kaplan-Meier sowie der Mantel-Cox-Log-Rank-Test eingesetzt. Die statistischen Berechnungen wurden mittels SPSS Statistics (IBM Corp. released 2023, IBM SPSS Statistics for Macintosh, Version 29.0.1.0. Armonk, NY: IBM Corp) durchgeführt. Ein Wert von *p* < 0,05 wurde als statistisch signifikant angesehen.

## Ergebnisse

### Übersicht

Eine Übersicht über die Patientenpopulation findet sich in Tab. 1. Hier verwendeten wir die bPSA-Gruppen 100–149 ng/ml, 150–249 ng/ml, 250–649 ng/ml sowie ≥ 650 ng/ml, um annähernd gleich große Vergleichsgruppen zu erhalten.

**Tab. 1:** Patientencharakteristika

			Gesamt	100–149 ng/ml	150–249 ng/ml	250–649 ng/ml	≥ 650 ng/ml
Betroffene	*n* (%)	–	695 (100)	183 (26,4)	159 (22,9)	181 (26,0)	172 (24,7)
Alter bei ED (Jahre)	Median	–	71	71	71	72	71
Baseline PSA (ng/ml)	Median	–	260	119,00	195,95	381,00	1417,00
5‑Jahres-Überlebensrate	% (SD)	–	68,5 (± 2,7)	76,9 (± 4,5)	77,0 (± 4,9)	61,4 (± 5,6)	57,4 (± 6,1)
Erster PSA-Nadir (ng/ml)	Median	–	3,35	1,38	2,29	5,14	10,38
Zeit bis zum ersten Nadir (Monate)	Median	–	6,77	7,23	7,27	6,61	6,04
PSA-Response erster Nadir	≤ 90 %	*n* (%)	145 (100)	38 (26,3)	29 (20,0)	44^c^ (30,3)	34^d^ (23,4)
	> 90 %	*n* (%)	545 (100)	145 (26,5)	130 (23,9)	134^c^ (24,6)	136^d^ (25,0)
Erster Nadir	< 0,2 ng/ml	*n* (%)	153 (100)	65 (42,5)	37 (24,2)	26^c^ (17,0)	25^d^ (16,3)
	≥ 0,2 ng/ml	*n* (%)	537 (100)	118 (22,0)	122 (22,7)	152^c^ (28,3)	145^d^ (27,0)
Jemals erreichter Nadir (ng/ml)	Median	–	2,10	0,66	1,43	4,79	4,84
Zeit bis jemals erreichter Nadir (Monate)	Median	–	8,98	9,17	9,21	8,15	9,08
PSA-Response jemals erreichter Nadir	≤ 90 %	*n* (%)	98	34 (34,7)	21 (21,4)	28 (28,6)	15 (15,3)
	> 90 %	*n* (%)	597	149 (25,0)	138 (23,1)	153 (25,6)	157 (26,3)
Jemals erreichter Nadir	< 0,2 ng/ml	*n* (%)	184	74 (40,2)	47 (25,5)	34 (18,5)	29 (15,8)
	≥ 0,2 ng/ml	*n* (%)	511	109 (21,3)	112 (21,9)	147 (28,8)	143 (28,0)
ISUP-Grade	Group 1 (GS ≤ 6)	*n* (%)	20	7 (35,0)	4 (20,0)	6 (30,0)	3 (15,0)
	Group 2 (GS 3 + 4 = 7)	*n* (%)	29	7 (24,2)	7 (24,1)	10 (34,5)	5 (17,2)
	Group 3 (GS 4 + 3 = 7)	*n* (%)	91	36 (39,5)	23 (25,3)	18 (19,8)	14 (15,4)
	Group 4 (GS 8)	*n* (%)	160	40 (25,0)	35 (21,8)	47 (29,4)	38 (23,8)
	Group 5 (GS 9–10)	*n* (%)	286	67 (23,4)	64 (22,4)	70 (24,5)	85 (29,7)
	n. a.	*n* (%)	109	26 (23,8)	26 (23,9)	30 (27,5)	27 (24,8)
UICC-Gruppen	UICC I + II^a^	*n* (%)	130 (100)	56 (43,1)	26 (20,0)	25 (19,2)	23 (17,7)
	UICC III + IV^b^	*n* (%)	479 (100)	106 (22,2)	108 (22,5)	141 (29,4)	124 (25,9)
	n. a.	*n* (%)	86 (100)	21 (24,4)	25 (29,1)	15 (17,4)	25 (29,1)

^a^T1-T2c N0 M0

^b^T3 N0 M0 + T4 oder jedes N1 und/oder M1

^c^Kein erster Nadir bei 3 Datensätzen dieser Gruppe

^d^Kein erster Nadir bei 2 Datensätzen dieser Gruppe

### Altersgruppen

**Abb. 2:**
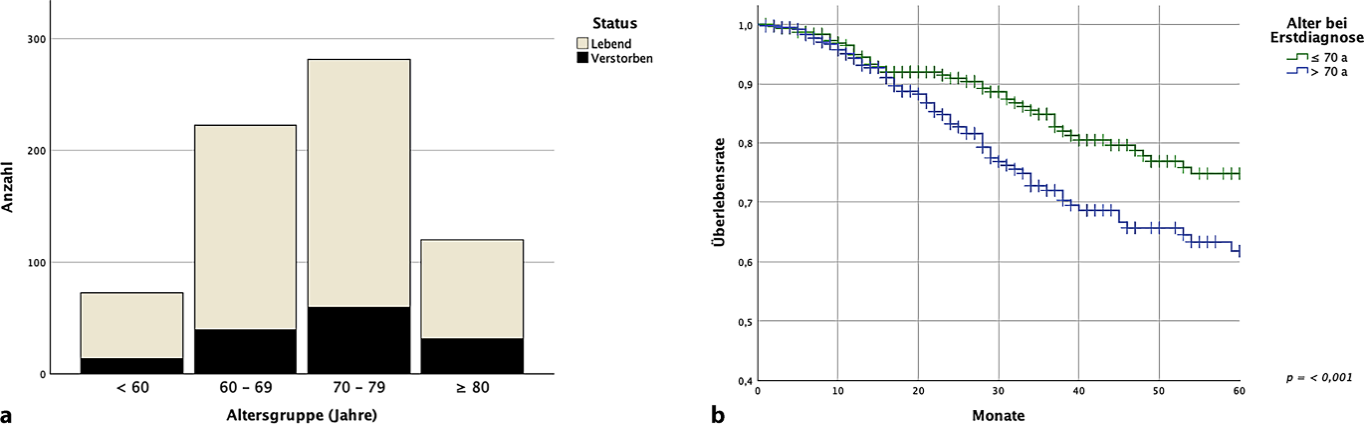
**a** Altersverteilung lebend/verstorben, **b** 5-Jahres-Gesamtüberleben (5-JGÜ) ≤ 70 Jahre /> 70 Jahre bei Erstdiagnose

Den Vergleich des Alters der Patienten bei Erstdiagnose führten wir anhand der Altersgruppen < 60, 60–69, 70–79 und ≥ 80 Jahre durch. Die höchste Inzidenz trat in der Gruppe der 70- bis 79-Jährigen auf. Ein Überblick über die Altersverteilung mit dem Anteil der zum Ende der Erhebung bereits verstorbenen Betroffenen findet sich in Abb. 2. Sofern das Alter bei Erstdiagnose ≤ 70 Jahre lag, zeigte sich ein hoch signifikanter Unterschied im 5‑JGÜ von 74,8 % (± 3,5 %), gegenüber dem 5‑JGÜ von 60,1 % (± 4,4 %) bei einem Alter von > 70 Jahren (*p* < 0,001).

### Überleben

**Abb. 3:**
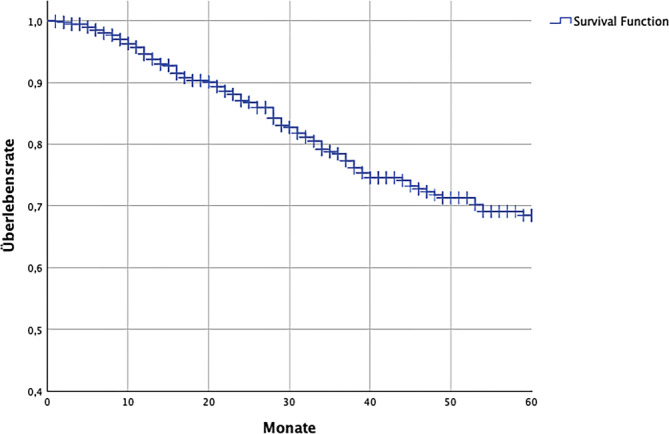
5‑Jahres-Gesamtüberlebensrate

Die Analyse nach Kaplan-Meier ergab ein medianes Überleben von 112 (± 9,8) Monaten, (95 %-KI 92,75–131,25 Monate). Bei Betrachtung der Überlebensfunktion zeigte sich, dass die Schätzung gegen Ende des Zeitraums zunehmend ungenau zu werden schien. Daher entschlossen wir uns, das 5‑JGÜ als bestmögliche Näherung an das reale Überleben anzugeben. Es zeigte sich in der Kohorte ein 5‑JGÜ von 68,5 % (± 2,7 %; Abb. 3). Der Status (lebend/verstorben) in der Kaplan-Meier-Analyse wurde durch die Angabe eines Todesdatums in der Patientenakte vergeben. Der Beobachtungszeitraum derjenigen, die kein dokumentiertes Todesdatum aufwiesen, wurde durch die Differenz zwischen dem Zeitpunkt der Erstdiagnose und dem Zeitpunkt des letzten Kontaktes („date last contact“) mit der dokumentierenden Einrichtung ermittelt.

### PSA-Betrachtungen

#### Baseline-PSA (bPSA)

**Abb. 4:**
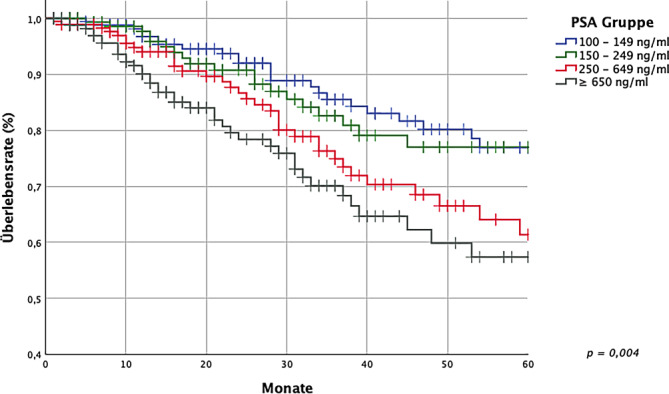
5‑Jahres-Überlebensrate nach bPSA-Gruppen (Baseline-PSA-Wert)

Der mediane bPSA-Wert zu Beginn der systemischen Therapie betrug 260,00 ng/ml. Der PSA-Wert zu Beginn der systemischen Therapie hatte einen signifikanten Einfluss (*p* < 0,001) auf die 5‑Jahres-Überlebensrate (5-JÜR). Die 5‑JÜR betrug 76,9 % ± 4,5 % in der Gruppe 100–149 ng/ml, 77,0 % ± 4,9 % in der Gruppe 150–249 ng/ml, 61,4 % ± 6,0 % in der Gruppe 250–649 ng/ml sowie 57,4 % ± 6,1 % in der Gruppe ≥ 650 ng/ml (Abb. 4). Diese Gruppen wurden rechnerisch anhand der Quartilgrenzen der bPSA-Werte ermittelt und gerundet, um die Lesbarkeit zu erhalten.

#### PSA-Nadir

**Abb. 5:**
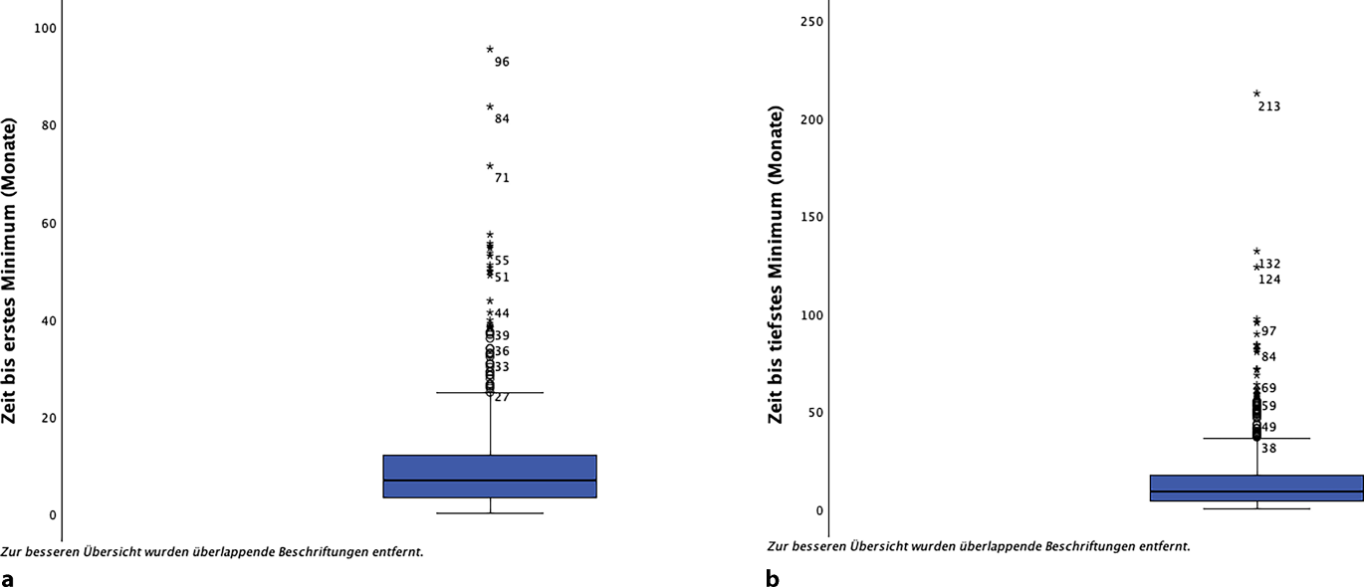
Boxplot: **a** Zeit bis ersten und **b** jemals erreichten Nadir (Monate)

Es wurden 2 verschiedene PSA-Tiefstwerte definiert: a) der erste Nadir nach Beginn der systemischen Therapie sowie b) der jemals erreichte Nadir über den gesamten Verlauf der Therapie. Hintergrund war die Beobachtung, dass im Verlauf der systemischen Therapie nach dem initialen Ansprechen häufig eine Intensivierung der Therapie durch den Einsatz von Kombinationen durchgeführt wurde. Hier stellte sich die Frage, ob auch das spätere Erreichen eines tiefen Nadirs Einfluss auf das 5‑JGÜ hat. In 0,2 % (*n* = 5) der Fälle wurde kein erster Nadir aufgezeichnet. Nach Beginn der Therapie wurde ein medianer erster PSA-Nadir von 3,35 ng/ml erreicht. Bei 22,2 % (*n* = 153) der Betroffenen wurde ein erster Nadir von < 0,20 ng/ml erzielt, 77,8 % (*n* = 537) erzielten einen ersten Nadir von ≥ 0,20 ng/ml. Wenn ein erster Nadir von < 0,20 ng/ml ermittelt wurde, zeigte sich ein hoch signifikanter Vorteil (*p* < 0,001) im 5‑JGÜ von 89,8 % (± 3,3 %), im Gegensatz zu der Situation, in der der erste Nadir ≥ 0,20 ng/ml betrug mit einem 5‑JGÜ von 60,4 % (± 3,5 %; Abb. 5). Der mediane jemals erreichte Nadir betrug 2,10 ng/ml. In 26,5 % (*n* = 184) der Fälle betrug der jemals erreichte Nadir < 0,2 ng/ml, in 73,5 % (*n* = 511) hingegen ≥ 0,2 ng/ml. Bei einem jemals erreichten Nadir von < 0,2 ng/ml zeigte sich ein hoch signifikanter Vorteil (*p* < 0,001) im 5‑JGÜ von 88,3 % (± 3,3 %), gegenüber einem 5‑JGÜ von 58,1 % (± 3,7 %) bei einem jemals erreichten Nadir von ≥ 0,2 ng/ml.

#### PSA-Response

In 79,0 % (*n* = 545) zeigte sich eine erste PSA-Response mit einem Abfall des bPSA-Wertes um > 90 % des bPSA-Wertes bezogen auf den ersten Nadir. In 21,0 % (*n* = 145) der Fälle lag die erste Response bei ≤ 90 %. Bei einer ersten PSA-Response von > 90 % zeigte sich ein hoch signifikanter Vorteil (*p* < 0,001) im 5‑JGÜ von 73,5 % (± 2,9 %) gegenüber einem 5‑JGÜ von 48,6 % (± 6,7 %), wenn die PSA-Response ≤ 90 % betrug (Abb. 5). Die jemals erreichte PSA-Response bezogen auf den jemals erreichten Nadir betrug bei 85,9 % (*n* = 597) der Betroffenen > 90 %, bei 14,1 % (*n* = 98) der Betroffenen ≤ 90 %. Eine jemals erreichte PSA-Response von > 90 % ergab einen hoch signifikanten Unterschied im 5‑JGÜ von 70,8 % (± 2,8 %) gegenüber einer jemals erreichten PSA-Response von ≤ 90 % bei der sich ein 5‑JGÜ von 48,8 % (± 10,2 %) ergab.

#### Zeit bis zum Nadir („time to nadir“, TTN)

**Abb. 6:**
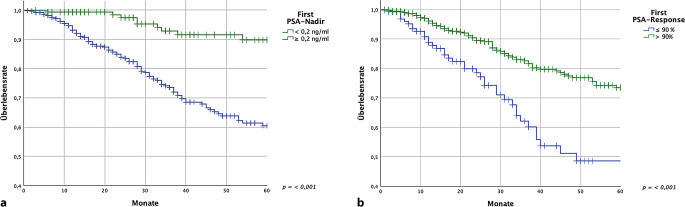
**a** 5-Jahres-Überlebensrate nach erstem PSA-Nadir und **b** erster PSA-Response (PSA ≥ 100 ng/ml)

Die Zeit bis zum ersten Nadir („time to first nadir“ – TTfN) betrug im Median 6,8 (± 10,6) Monate (Abb. 6). Die Zeit bis zum jemals erreichten Nadir („time to overall nadir“ – TToN) betrug im Median 9,0 (± 18,2) Monate. Hierbei zeigte sich in der Gruppe der Betroffenen mit einem ersten Nadir von < 0,2 ng/ml kein Einfluss einer TTfN von ≥ 6 Monaten auf das 5‑JGÜ. In der Gruppe derjenigen, die keinen PSA-Nadir von < 0,2 ng/ml erreichten, zeigte sich jedoch ein signifikanter Vorteil bezüglich des 5‑JGÜ, wenn die TTfN ≥ 6 Monate betrug.

### Gleason-Score

In 84,3 % (*n* = 586) der Fälle lagen uns valide und somit für den Vergleich verwertbare Ergebnisse der histopathologischen Untersuchung vor. Am häufigsten wurden die Muster 4 + 5 = 9 in 32,1 % (*n* = 188) und 4 + 4 = 8 in 25,8 % (*n* = 151) der Fälle angegeben. Zur Betrachtung der Wertigkeit des Gleason-Score unterteilten wir diese 586 Patienten entsprechend der ISUP-Grade (International Society of Urological Pathology; [9, 10, 18]). In nahezu der Hälfte der Fälle 48,8 % (*n* = 286) trat ein Grad 5 auf, gefolgt von Grad 4 mit 27,3 % (*n* = 160), Grad 3 mit 15,5 % (*n* = 91), Grad 2 mit 4,9 % (*n* = 29) und Grad 1 mit 3,4 % (*n* = 20). Bei 15,7 % (*n* = 109) wurde kein Gleason-Score angegeben. Das 5‑JÜR für Patienten mit einem Prostatakarzinom des ISUP-Grades 5 betrug 59,3 % (± 4,7), für ISUP-Grad 4 69,9 % (± 5,7), für ISUP-Grad 3 78,7 % (± 7,7), für ISUP-Grad 2 75,2 % (± 8,9) sowie für ISUP-Grad 1 71,7 % (± 10,8).

### Staging

Die ermittelten TNM-Stadien fassten wir anhand der UICC-Stadien zusammen. In 87,6 % (*n* = 609) Fällen lagen uns Informationen zu den UICC-Stadien vor. In hiervon 71,3 % (*n* = 434) der Fälle trat das UICC-Stadium IV auf. Stadium I zeigte sich in 16,1 % (*n* = 98) der Fälle, gefolgt von Stadium III in 7,4 % (*n* = 45) sowie Stadium II in 5,3 % (*n* = 32) der Fälle.

### Metastasierung

Bei 80,4 % (*n* = 559) der Patienten wurde eine auswertbare Angabe zur Metastasierung dokumentiert. In 472 Fällen wurde auch der Ort der Metastasierung in den Kategorien Knochenmetastasen, Organmetastasen und viszerale Metastasen dokumentiert. Hiervon traten mit 66,3 % (*n* = 313) am häufigsten alleinige Knochenmetastasen auf, gefolgt von 26,3 % (*n* = 124) der Betroffenen, die eine Kombination aus Metastasen aller drei Kategorien aufwiesen. Deutlich seltener trat eine Kombination aus Organmetastasen und viszeralen Metastasen in 6,6 % (*n* = 31) der Fälle auf. Weitere Kombinationen traten noch seltener auf. Eine Einteilung der Metastasenlast nach den CHAARTED-Kriterien war anhand der vorliegenden Daten in 35,0 % (*n* = 291) Fällen möglich. Diesen Kriterien folgend lag bei diesen Betroffenen in 83,5 % (*n* = 243) der Fälle eine hohe Metastasenlast („high volume“), bei 16,5 % (*n* = 48) der Betroffenen eine niedrige Metastasenlast („low volume“) vor [31].

### Therapie

Im Folgenden werden die angegebenen medikamentösen und operativen Therapien dargestellt.

#### Initiale Therapie

Als initiale Therapie wurde bei 86,2 % (*n* = 599) der Betroffenen eine medikamentöse antihormonelle Therapie dokumentiert. Mit einer Häufigkeit von 1,3 % (*n* = 9) fand sich als eine chirurgische antihormonelle Therapie eine Orchiektomie. Bei 8,2 % (*n* = 57) der Betroffenen wurde als initiale Therapie eine radikale Prostatektomie festgehalten. Seltener wurde als initiale Therapie eine Chemotherapie in 2,3 % (*n* = 16) sowie eine externe Strahlentherapie der Prostata in 1,6 % (*n* = 11) der Fälle angegeben. Bei hiervon 0,8 % (*n* = 6) der Betroffenen wurde eine solitäre Strahlentherapie ohne weitere systemische Therapien angegeben, bei 0,7 % (*n* = 5) der Betroffenen eine initiale Kombination aus Strahlentherapie und antihormoneller Therapie. In 0,3 % (*n* = 2) aller Fälle wurden „andere Therapien“ erfasst, die nicht näher klassifiziert waren, in 0,1 % (*n* = 1) aller Fälle wurde die Teilnahme an einen Studienprotokoll angegeben.

#### Systemische Erstlinientherapie

##### Monotherapien.

In etwa der Hälfte der Fälle 49,4 % (*n* = 343) wurde als systemische Erstlinientherapie eine Monotherapie mit einem Gonadotropin-Releasing-Hormon (GnRH) -Agonisten oder Antagonisten angegeben. Die kurzzeitige Anwendung eines Antiandrogens für 4–6 Wochen zu Beginn der Therapie mit einem GnRH-Agonisten, auch als „Flare-Prophylaxe“ bezeichnet, wurde in 29,4 % (*n* = 101) der zuvor genannten Fälle dokumentiert [32]. In 11,9 % (*n* = 83) der Fälle hingegen findet sich eine Monotherapie mit einem Antiandrogen. In 1,2 % (*n* = 8) der Fälle ist die Substanzgruppe der Hormontherapie nicht angegeben. Weiterhin finden sich Angaben zu Monotherapien die aus der Anwendung einer Chemotherapie in 2,4 % (*n* = 17) sowie der Anwendung von Abirateron/Prednisolon als Einzelsubstanz in 1,2 % (*n* = 8) der Fälle bestanden.

##### Kombinationstherapien.

Die dokumentierten Kombinationstherapien können in zwei Gruppen gegliedert werden. Eine davon stellt die komplette Androgenblockade (CAB) mit der dauerhaften Kombination eines GnRH-Agonisten oder Antagonisten mit einem Antiandrogen in 25,8 % (*n* = 179) der Fälle dar. Die andere mit 2,6 % (*n* = 18) deutlich kleinere Gruppe umfasst alle weiteren erfassten Kombinationen.

#### Zweitlinientherapie

In 42,3 % (*n* = 294) der Fälle wurde eine systemische Zweitlinientherapie angegeben. Dabei fanden sich in 38,4 % (*n* = 113) dieser Fälle Substanzen aus der Gruppe der Taxane, vornehmlich Docetaxel, dicht gefolgt von Abirateron/Prednisolon in 37,1 % (*n* = 109) der Fälle. Die neuartigen hormonellen Substanzen (NHA) Enzalutamid und Apalutamid, auch als Androgensignalweginhibitoren (ASI) bezeichnet, zeigten sich in 11,9 % (*n* = 35) der Fälle bezogen auf Enzalutamid und in 7,8 % (*n* = 23) der Fälle bezogen auf Apalutamid. Weitere Substanzen sind deutlich seltener zu finden.

#### Drittlinientherapie

In 17,6 % (*n* = 122) der Fälle wurde eine systemische Drittlinientherapie dokumentiert. Hiervon finden sich bei 41,0 % (*n* = 50) der Patienten Abirateron/Prednisolon sowie bei 26,2 % (*n* = 32) der Patienten Enzalutamid. In 24,6 % (*n* = 30) der Fälle wurde eine Chemotherapie mit Taxanen, vornehmlich Docetaxel, hinterlegt. Weitere Substanzen finden sich deutlich seltener.

**Abb. 7:**
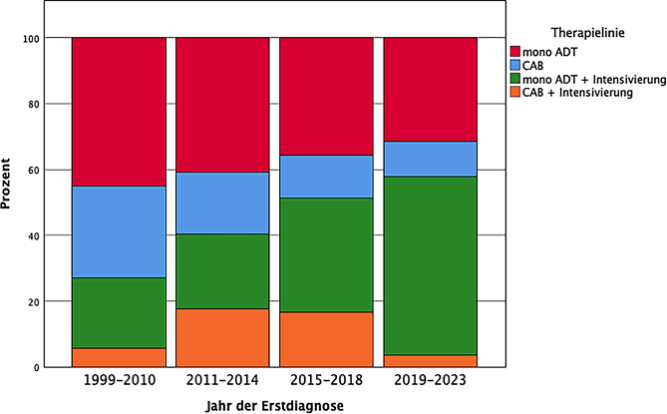
Zeitlicher Verlauf der medikamentösen Therapielinien (gesamte Kohorte)

#### Weitere Therapieansätze

Bei fehlendem Ansprechen oder Therapiewechsel aus anderen Gründen wurden nach der Drittlinientherapie in 9,5 % (*n* = 66) der Fälle noch weitere Therapieansätze angegeben. Hiervon kamen aus Sicht der medikamentösen Therapie vor allem Enzalutamid und eine Chemotherapie aus der Gruppe der Taxane bei jeweils 31,8 % (*n* = 21) der Patienten vor. Abirateron/Prednisolon wurde bei 9 % (*n* = 6) als weitere Therapie angegeben. Darüber hinaus zeigte sich ein sehr heterogenes Bild an selteneren Therapieansätzen.

#### Strahlentherapie (ST)

Bei 34,1 % (*n* = 237) der Patienten wurde über den gesamten Zeitraum ihrer Therapie ein strahlentherapeutisches Verfahren erfasst. Davon wurde bei 4,6 % (*n* = 11) dieser Betroffenen eine externe Strahlentherapie als initiale Therapie angegeben, wovon bei 54,5 % (*n* = 6) keine weitere Therapie, bei 45,5 % (*n* = 5) zusätzlich eine zeitnahe antihormonelle Therapie angegeben wurde. In den restlichen 95,4 % (*n* = 226) der Fälle, in denen ein strahlentherapeutisches Verfahren dokumentiert wurde, wurde dieses im Verlauf der Therapie der Erkrankung erfasst. Bei allen strahlentherapeutischen Verfahren (*n* = 237) wurde zudem die Intention der Behandlung dokumentiert. Dabei wurden einzelne Intentionen sowie kombinierte Intentionen angegeben. Als Beispiel für eine Kombination von Intentionen könnte eine initial kurativ intendierte ST gelten, auch im Sinne einer „Salvage-Radiotherapie“, die dann um eine ADT ergänzt wird, die wiederum zu einer symptomatischen Gynäkomastie führen kann, woraufhin eine Bestrahlung der Mamillen in einigen Fällen angeboten werden kann. Tritt im weiteren Verlauf der Erkrankung dann eine Knochenmetastase der Wirbelsäule auf, wird diese in palliativer Intention bestrahlt. Hieraus ergibt sich dann die Kombination aus kurativer Intention, Bestrahlung der Mamillen und schließlich palliativer Bestrahlung der Metastase.

Bei 77,6 % (*n* = 184) der Betroffenen, die ein strahlentherapeutisches Verfahren erhielten, wurde eine einzelne Intention angegeben, die Verteilung hierbei war wie folgt: 50,5 % (*n* = 93) kurative Intentionen, 40,8 % (*n* = 75) palliative Intentionen, 8,7 % (*n* = 16) Bestrahlungen der Mamillen. Bezüglich der kombinierten Intentionen wurde bei 12,2 % (*n* = 29) Betroffenen eine initial kurative, im Verlauf dann palliative Intention angegeben. Bei wiederum 4,7 % (*n* = 11) der Betroffenen wurde eine Bestrahlung der Mamillen neben einer kurativ intendierten ST dokumentiert. Weiterhin wurde bei 3,8 % (*n* = 9) der Betroffenen neben einer palliativ intendierten Bestrahlung eine Bestrahlung der Mamillen angegeben. Die seltenste Kombination war die Kombination aus den Intentionen kurativ und palliativ sowie einer Bestrahlung der Mamillen bei 1,7 % (*n* = 4) Betroffenen.

## Diskussion

Der PSA-Wert dient als diagnostischer Wegweiser, sei es im Rahmen der Früherkennung, des Screenings von Hochrisikogruppen, zur Kontrolle des Therapieansprechens oder auch bei der Detektion eines Rezidivs nach definiter Therapie in kurativer Intention. Im niedrig ein- bis zweistelligen Bereich erlaubt der Stand der aktuellen Forschung, situationsbezogene PSA-Grenzwerte und daraus folgende evidenzbasierte Handlungsempfehlungen festzulegen. Jedoch werden Betroffene und Behandelnde auch mit PSA-Werten konfrontiert, die weit über diesen Bereich hinausgehen. Liegt der PSA-Wert in dem Extrembereich von weit jenseits der 100 ng/ml, steigt auch die Unsicherheit über die Bedeutung dieser Erhöhung. Ist sie Ausdruck einer infausten Prognose und hat sie unmittelbaren Einfluss auf das Therapieziel – die Therapieauswahl? Was kann eine Therapie in dieser Situation noch leisten?

Um diesen Fragen nachzugehen, untersuchten wir Real-world-Daten von 695 Betroffenen aus der ambulanten und stationären Versorgung, die sich für eine Therapie entschieden hatten. In dieser Analyse legten wir den Fokus auf eine Betrachtung des Überlebens und der angewendeten Therapien bei extremen PSA-Werten. Bei einem medianen bPSA-Wert von 260 ng/ml zeigte sich ein unerwartet hohes 5‑JÜR von 68,5 % trotz prognostisch negativer Vorzeichen wie einer bestehenden Metastasierung in 76 % der Fälle oder einem hohen Gleason-Score. Selbst in der Altersgruppe von > 70 Jahren wurde ein 5‑JÜR von 60 % erreicht.

Aus diesen Ergebnissen kann abgeleitet werden, dass trotz extremer PSA-Werte sehr viele Patienten von einer Therapie und ihrer möglichen Intensivierung profitieren – auch wenn die Situation palliativ bleibt. Unter diesem Aspekt müssen die Therapieziele gemeinsam mit dem Patienten definiert werden und die Prognose realistisch abgeschätzt werden. Das Ergebnis dieser Untersuchung kann dabei sicher helfen.

Gerade zu Beginn der systemischen Therapie bei extremen PSA-Werten ist die Frage wichtig, ob es Anzeichen für ein gutes Therapieansprechen und damit eine bessere Prognose gibt.

Hier sehen wir klare Hinweise, dass sowohl ein erster Nadir als auch ein jemals erreichter Nadir von < 0,2 ng/ml zu einer signifikanten Erhöhung des 5‑JÜR führen. Dies gilt ebenso für eine erste, als auch eine insgesamte PSA-Response von > 90 %.

Diese Ergebnisse sprechen dafür, dass die prognostische Relevanz eines niedrigen Nadirs auch im extremen bPSA-Bereich zu bestehen scheint. Ein therapeutisches Ziel sollte daher das Erreichen eines tiefen Nadirs sein. Durch die Betrachtung des jemals erreichten Nadirs konnten wir sehen, dass auch das spätere Erreichen eines tiefen Nadirs, beispielsweise durch die Erweiterung der initialen Therapie, zu einer signifikanten Verlängerung des 5‑JÜR beitragen kann.

In den Daten der ARASENS-Studie zeigte sich ebenfalls, dass das Erreichen eines Nadirs von < 0,2 ng/ml unter der Therapie mit ADT, Docetaxel und Darolutamid einen signifikanten Vorteil bezüglich des insgesamten Überlebens (OS) bietet [28].

Auch in den Daten aus der CHAARTED-Studie zeigt sich, dass das Erreichen eines tiefen Nadirs von ≤ 0,2 ng/ml nach 7 Monaten unter einer ADT mit oder ohne Docetaxel bei einem metastasierten hormonsensitiven PCa (mHSPCa) einen deutlichen Überlebensvorteil bringt [13]. Dies gilt ebenso für die TITAN-Studie, in der für die Kombination von ADT und Apalutamid ein Überlebensvorteil bei einem PSA-Nadir von ≤ 0,2 ng/ml ergab [24]. Dies stimmt insofern mit unserer Untersuchung überein, dass auch in unserer Kohorte ein Vorteil bezüglich des Überlebens bei einem ersten Nadir von < 0,2 ng/ml besteht und die mediane TTN (hier als Zeit bis zum ersten Nadir bezeichnet) etwa 7 Monate betrug. Ein Übersichtsartikel von Halabi et al. kommt ebenfalls zu dem Ergebnis, dass ein PSA von ≤ 0,2 ng/ml einen Überlebensvorteil für Betroffenen mit einem mHSPCa bietet [12].

Bezüglich einer tiefen PSA-Response von ≥ 90 % (PSA90) bei Betroffenen mit einem mHSPCa konnte aus Untersuchungen wie der TITAN sowie SPARTAN (Apalutamid + ADT) und LATITUDE (Abirateron + ADT) gezeigt werden, dass ein positiver Effekt auf progressionsfreies sowie metastasenfreies Überleben, respektive Gesamtüberleben, besteht [6, 23]. In unserer Untersuchung wurde, verglichen mit den genannten Untersuchungen, häufiger eine insgesamte PSA-Response von ≥ 90 % (PSA90) erreicht. In einer Real-world-Untersuchung von Lowentritt et al. (2023) an einer ambulanten nordamerikanischen Kohorte (*n* = 364) wurde ebenfalls bei deutlich weniger Betroffenen eine PSA90 erreicht [20]. Hier ergab sich für Apalutamid eine PSA90 von 66,2 % sowie eine PSA90 von 43,4 % für Abirateron nach 6 Monaten [20]. Unsere Untersuchung kann einen Hinweis darauf geben, dass Betroffene mit extremen PSA-Werten ein gutes Therapieansprechen aufweisen und von einer Therapie profitieren können, was einen relevanten Aspekt im Austausch mit Betroffenen darstellt. Zudem kann dieses Ergebnis als Ausgangspunkt für weitere Untersuchungen bezogen auf konkrete, medikamentöse Therapiestrategien bei Betroffenen mit extremen PSA-Werten genutzt werden.

Die Zeit bis zum ersten Nadir betrug in dieser Kohorte im Median 6,8 (± 10,6) Monate (Abb. 6). Die Zeit bis zum jemals erreichten Nadir betrug im Median 9,0 (± 18,3) Monate. Diese kurze Zeit bis zum Nadir ist ein Hinweis auf das starke Ansprechen der Therapie in dieser Erkrankungssituation. Dieser „schnelle“ Effekt kann jedoch dazu führen, dass eine Intensivierung nicht mehr als nötig erachtet wird. Dem gegenüber wirft die Analyse des „jemals erreichten Nadirs“ die Frage auf, ob eine spätere Intensivierung der Therapie nicht dennoch zusätzlich Vorteile birgt. Bezüglich der prognostischen Relevanz der Zeit bis zum Nadir und ihrer Bedeutung für das Gesamtüberleben, kamen bisherige Untersuchungen zu unterschiedlichen Ergebnissen. Aktuell scheint sich, bei Betroffenen mit einem mHSPCa, eine längere Zeit bis zum Nadir positiv auf das Gesamtüberleben auszuwirken. Hierbei variieren die Grenzwerte zwischen ≥ 6 oder ≥ 12 Monaten [14, 29]. In der Subgruppe von Betroffenen mit einem PSA-Nadir von ≤ 0,2 ng/ml scheint das jedoch nicht zu gelten [7, 8]. Diesen Trend sehen wir ebenfalls in unseren Daten, mit den Limitationen des retrospektiven Studiendesigns. Das Erreichen eines tiefen Nadirs < 0,2 ng/ml scheint somit prognostisch relevanter zu sein als die Zeit bis zum Erreichen dieses tiefen Nadirs [17]. Dies unterstützt die Hypothese, dass auch das spätere Erreichen eines tiefen Nadirs durch eine Intensivierung der Therapie positive Effekte haben könnte.

Nachdem es sich hier um die Betrachtung von Real-world-Daten handelt, können wir keine Aussagen bezüglich der Effektivität einzelner Therapieregime machen. Das Ziel der Darstellung der verschiedenen Therapiestrategien im zeitlichen Verlauf liegt vielmehr darin, einen Überblick über die in dieser Patientenpopulation angewendeten Therapien zu geben.

So können wir eine Entwicklung in den Therapiestrategien sehen. Es zeigte sich ein zeitlicher Wandel von ADT-Monotherapien über frühe Kombinationstherapien, wie der kompletten Androgenblockade, und gestaffelte Kombinationstherapien, im Sinne von aufeinander aufbauenden Therapielinien, hin zu tatsächlichen initialen Kombinationstherapien (Abb. 7). Eine solche Kombination kann beispielsweise aus der Kombination einer ADT mit einem Wirkstoff aus der Gruppe der neuartigen hormonellen Substanzen (NHA) oder einer Chemotherapie aus der Gruppe der Taxane bestehen. Diese initialen Kombinationen aus mehreren Substanzgruppen sind in unserer Kohorte noch selten anzutreffen. Hier zeigt sich eine Diskrepanz zwischen den Empfehlungen der aktuellen S3-Leitlinie und der therapeutischen Realität [19]. Bei Betrachtung der gesamten Kohorte, ist hierbei natürlich zu bedenken, dass die Daten in einem Zeitraum von 1999 bis 2023 erhoben wurden. In den Jahren 2019 bis 2023 sind die Intensivierungen einer initialen ADT mit NHA oder einer Chemotherapie deutlich dominanter. In unserer Kohorte wird eine Intensivierung annähernd gleich häufig mittels einer Chemotherapie mit Docetaxel sowie einer Therapie mit dem NHA Abirateron als Zweitlinientherapie durchgeführt. Deutlich seltener kommt eine Drittlinientherapie zur Anwendung, hierbei haben die NHA mit Abirateron und Enzalutamid die Oberhand gegenüber der Chemotherapie mit Docetaxel. Wie aus diesen Ergebnissen zur Zweit- und Drittlinientherapie deutlich wird, kam es über den Zeitraum der Betrachtung dieser Untersuchung zu vielgestaltigen Veränderungen in der Therapielandschaft.

Auch in anderen Untersuchungen zum internationalen Vergleich von Real-world-Daten lassen sich diese Unterschiede zwischen der therapeutischen Realität und den Leitlinienempfehlungen deutlich belegen. Hier zeigt sich ebenfalls ein Trend zur Intensivierung der Therapie mittels NHA oder Chemotherapie seit 2021, v. a. bei Betroffenen die an einem mHSPCa erkrankt sind und u. a. eine hohe Krankheitslast bei einer hohen Restlebenserwartung sowie einer geringe Beeinträchtigung durch Grunderkrankungen aufweisen [25]. Dabei zeigt sich für eine Intensivierung eine Dominanz der NHA über der Chemotherapie bezüglich der Intensivierung [25]. Dennoch spielt die Mono-ADT weiterhin eine große Rolle [25]. In einer Untersuchung von Real-world-Versorgungsdaten aus den Zentren des DVPZ – jetzt Uro-Cert – mittels der UroCloud aus dem Jahr 2015 konnte ebenfalls der verminderte Einsatz von Kombinationstherapien bei primärer Hormontherapie, trotz bereits zu dieser Zeit anderslautender Empfehlungen, gezeigt werden [3].

Inzwischen gibt es durch die PEACE-1- sowie die ARASENS-Studien klare Hinweise darauf, dass die Dreifachkombinationen aus ADT, Docetaxel und Abirateron/Prednisolon, respektive ADT, Docetaxel und Darolutamid das Risiko, zu versterben gegenüber der Zweifachkombination aus ADT und Docetaxel senken können [11, 21, 30]. Hier wird sich zeigen, inwieweit diese Dreifachkombinationen in der Zukunft bei Betroffenen mit extremen PSA-Werten zunehmend Anwendung finden.

Die Gründe für die fehlende Intensivierung oder fehlende initiale Kombinationstherapien können vielschichtig sein und sind durch diese Studie nicht zu ermitteln. Hier besteht weiterer Forschungsbedarf hinsichtlich der Frage, welche Hindernisse in der Patientenversorgung außerhalb von RCT auftreten, die zum selteneren Einsatz von Doppel- oder Dreifachtherapien führen. Ein Trend zur Intensivierung der Therapien mittels NHA und Chemotherapie zeichnet sich bereits ab.

Ein möglicher Grund für das hier erkennbare, überraschend lange 5‑JÜR besonders der sehr hohen PSA-Werte könnte sein, dass im Angesicht der extremen PSA-Werte, auch ohne die Evidenz zur Effektivität der Doppel- oder Dreifachkombinationen, in dieser „historischen“ Kohorte die Intensivierung der Therapie als einzige Chance auf ein gutes Ansprechen gesehen wurde.

Zusammenfassend sehen wir klare Hinweise darauf, dass medikamentöse Therapien auch im extremen PSA-Bereich Anwendung finden können und bei entsprechendem Therapieziel auch Anwendung finden sollten.

Nach unserem Wissen stellt diese Untersuchung eine der ersten Real-world-Untersuchungen in der deutschsprachigen Uroonkologie dar, die das Schicksal von Patienten mit extremen PSA-Werten zum Gegenstand hat und die Therapieoptionen in dieser Erkrankungssituation untersucht.

## Limitationen

Der retrospektive Charakter der Untersuchung stellt eine klare Limitation dar. Wie bei allen Untersuchungen von Real-world-Daten besteht das Problem, dass einzelne Datenelemente zum Zeitpunkt der Erfassung nicht als relevant bekannt waren und sie deshalb auch nicht in der Datengüte vorliegen, wie sie beispielsweise für die Analyse kausaler Zusammenhänge benötigt würden. Darüber hinaus liegt es auch in der Natur anonymisierter Daten, das eine „source data verification“ nicht durchgeführt werden kann und so ebenfalls eine weitere Hürde für die Datentiefe darstellt.

Ein mögliches Confounding ist zudem nicht auszuschließen, da nicht alle Zentren einheitlich – wie beispielsweise in einem gemeinsamen Studienprotokoll – dokumentieren, was möglicherweise einen „selection bias“ nach sich zieht. Die Ergebnisse dieser Studie sollten daher als Überblick über die aktuelle Therapielandschaft in dieser Erkrankungssituation gesehen werden und kann Ausgangspunkt für prospektive, gegebenenfalls randomisierte Analysen bieten, um die hier gemachten Hypothesen zu untermauern.

Durch das primäre Einschlusskriterium PSA ≥ 100 ng/ml werden in dieser Untersuchung sowohl Betroffene betrachtet, die bereits im Vorhinein eine lokale Therapie erhalten haben und ein biochemisches Rezidiv erleiden, als auch diejenigen, die sich vor einer primären systemischen Therapie befinden. Hierdurch entsteht eine Inhomogenität der Kohorte, die auch dazu führt, dass keine belastbaren Aussagen über das Therapieansprechen der lokalen Therapien getroffen werden konnten. Dies wurde in Kauf genommen, um die Versorgungsrealität derjenigen abzubilden, die unabhängig von der Vortherapie einen extremen PSA-Wert aufweisen. Der retrospektive Charakter dieser Datenbankanalyse lässt es zudem nicht zu, die Einflussfaktoren auf Therapieentscheidungen nachzuvollziehen, die zu den beschriebenen Therapieverläufen geführt haben. Der schnelle Wandel der therapeutischen Optionen in der metastasierten Situation dürfte zusätzlich dazu beitragen, dass generalisierbare Aussagen zum Gesamtverlauf zum jetzigen Zeitpunkt nicht extrapoliert werden können.

Durch die Berechnung des Beobachtungszeitraums mittels des Zeitpunkts des letzten Kontakts („date last contact“) in den Fällen, in denen kein Todesdatum bekannt ist, kann es zu einer Unterschätzung des Überlebens gekommen sein. Es wurde keine Auswertung von Sterberegisterdaten vorgenommen, da die analysierten Datensätze anonymisiert vorlagen.

## Fazit für die Praxis

- Die vorliegenden Daten zeigen, dass eine Therapie bei Betroffenen mit extremen PSA-Werten durchaus langfristig Erfolge erzielen kann und ein extremer PSA-Wert für sich kein Ausdruck einer ungünstigen Prognose ist.
- Eine medikamentöse Therapie hat im extremen PSA-Bereich Erfolgsaussichten.
- Wenn möglich sollte eine Intensivierung respektive Kombination von Therapien erfolgen.
- Das Ziel sollte das Erreichen eines tiefst möglichen Nadirs oder einer PSA-Response von ≥90 % sein.
- Auch das Erreichen eines späteren tiefen Nadirs könnte einen Überlebensvorteil bieten.

## Data Availability

Die erhobenen Datensätze können auf begründete Anfrage in anonymisierter Form beim korrespondierenden Autor angefordert werden. Die Daten befinden sich auf einem Datenspeicher am Uniklinikum Erlangen.
